# News and Events of the Week

**Published:** 1911-06-03

**Authors:** 


### News and Events of the Week.

The date originally fixed for the meeting in Rome of the seventh Congress of dermatology and syphiligraphy has been changed to September 18 next, and will last until the 23rd. Dr. Ciarrochi, of 5 Piazza Grazioli, Rome, is the general secretary.

The second International Congress of Urology will be held in London from July 24 to 28 next under the presidency of Professor Harry Fenwick. The subjects put down for discussion are as follows: Phosphaturia and oxaluria; the late effects of prostatectomy; extensive resection of the bladder. Dr. Desnos, of 59 rue de la Boëtie, Paris, is the general secretary.

The Royal Insurance Company have forwarded a copy of their extremely useful and handy "Record of Sports." This publication is now in its eighth annual issue, and extends to 256 pages, crammed with records and record-holders of every imaginable sport in which the British race indulges. This year aviation receives notice; in connection with this new form of human enterprise a comprehensive series of notable performances and events is included. The Royal Insurance Company, whose address is 1 North John Street, Liverpool, will be happy to supply any reader of The Hospital with a free copy of this interesting reference-book until the stock of them is exhausted. Application should be made direct to the company, not through the offices of this paper.

According to the latest returns of the Registrar-General, 94,828 infants under one year of age died last year in England and Wales, and out of a total of half a million deaths, one in every five was that of a baby. And by far the largest proportion of these deaths was attributed to digestive troubles, caused, undoubtedly, by improper feeding. Milk is, or should be, the one and only food of infants, and unless it is supplied pure and kept pure in the home, great danger may arise from its use. To promote a clean milk supply, a series of leaflets on the subject has been published by the National League for Physical Examination and Improvement. We are asked to state that, in view of the approaching hot weather, free copies will be sent to all who apply to the Secretary of the League, at 4 Tavistock Square, W.C., if postage is enclosed.

The eighth voyage for purposes of scientific and medical improvement organised by "l'enseignement médico-mutuel international" (the English branch of which is in process of formation) will take place from August 12 to August 30 in Scandinavia (Denmark, Norway, and Sweden). The following itinerary has been arranged: Hamburg, Altona, Kiel, Korsor, Copenhagen, Skodsborg, Klampendorg, Copenhagen, Elsinore, Gottborg, the falls of Froloetan, Christiania, Myrdal, Flaam, the Sognefjord, Gudwangen, Stalheim, Voss, Bergen, the Hardangerfjord, Odde, Eide, Christiania, Holmenkollen, Friksta, Hoping, Stockholm, Upsala, Lake Moelar, Katrineholm, Mjolby, Lund, Trelleborg, Sassnitz, Stralsund, Berlin. The tour will end at Berlin at the moment when the International Congress of oto-rhino-laryngology and the Congress of the "Goultes de lait" are in progress. At the same time the International Exhibition of Hygiene will be in full swing at Dresden.

A banquet of the Brussels Medical Graduate Association will be held at the Garden Club of the Coronation Exhibition on Thursday, June 8, at eight o'clock. All graduates of the University of Brussels are welcome, and members are invited to bring ladies. Tickets 7s. 6d. each (not including wine) may be obtained from the Honorary Secretary, Dr. Arthur Haydon, 23 Henrietta Street, Cavendish Square.

The Executive Committee of the Florence Nightingale Memorial, of which Lord Crewe is chairman and Lord Pembroke deputy-chairman, have received the following donations: The Duke of Fife, £10 10s.; Lord Pembroke, £50; Lord Salisbury, £25; Lord Lansdowne, £5; Lady Wantage, £100; Sir John Wolfe Barry, £10 10s.

The decision of the Sheriff's Substitute at Dunfermline in the action brought by Mr. Gordon, a chemist and druggist, against Dr. Sanjana, of Kelty, for damages in respect of an alleged slander has been reversed. The case before the Under-Sheriff was reported in a recent issue of The Hospital, and it will be remembered that the Sheriff Substitute awarded Mr. Gordon £30 damages.

The New Palace Steamers' *Royal Sovereign* will commence her summer sailings down the Thames to Southend, Margate, and Ramsgate on Saturday, June 3, and her sister ship, the *Koh-i-Noor*, to Deal and Dover on Sunday, June 18. Both the steamers have been thoroughly overhauled during the winter months, and have passed all the requirements of the Board of Trade for their passenger certificates. The Saturday afternoon trips to Margate by the *Koh-i-Noor*, commonly known as the "Husbands' Boat," will commence on June 17, and continue throughout the season. The circular bookings with the South-Eastern and Chatham Railway, down by boat and home by rail, which have proved so popular in past years, have again been arranged for, but with greater facilities, the 8s. 6d. first saloon and third rail ticket being now available for fifteen days instead of from Saturday to Monday, as formerly.

